# Characterization of Increased Extracellular Vesicle-Mediated Tigecycline Resistance in *Acinetobacter baumannii*

**DOI:** 10.3390/pharmaceutics15041251

**Published:** 2023-04-15

**Authors:** Hyejin Cho, Tesalonika Sondak, Kwang-sun Kim

**Affiliations:** Department of Chemistry and Chemistry Institute of Functional Materials, Pusan National University, Busan 46241, Republic of Korea

**Keywords:** *Acinetobacter baumannii*, antimicrobial resistance, extracellular vesicles, tigecycline, bacterial infection

## Abstract

Carbapenem-resistant *Acinetobacter baumannii* (CRAB) is the most detrimental pathogen that causes hospital-acquired infections. Tigecycline (TIG) is currently used as a potent antibiotic for treating CRAB infections; however, its overuse substantially induces the development of resistant isolates. Some molecular aspects of the resistance mechanisms of AB to TIG have been reported, but they are expected to be far more complicated and diverse than what has been characterized thus far. In this study, we identified bacterial extracellular vesicles (EVs), which are nano-sized lipid-bilayered spherical structures, as mediators of TIG resistance. Using laboratory-made TIG-resistant AB (TIG-R AB), we demonstrated that TIG-R AB produced more EVs than control TIG-susceptible AB (TIG-S AB). Transfer analysis of TIG-R AB-derived EVs treated with proteinase or DNase to recipient TIG-S AB showed that TIG-R EV proteins are major factors in TIG resistance transfer. Additional transfer spectrum analysis demonstrated that EV-mediated TIG resistance was selectively transferred to *Escherichia coli*, *Salmonella typhimurium*, and *Proteus mirabilis*. However, this action was not observed in *Klebsiella pneumonia* and *Staphylococcus aureus*. Finally, we showed that EVs are more likely to induce TIG resistance than antibiotics. Our data provide direct evidence that EVs are potent cell-derived components with a high, selective occurrence of TIG resistance in neighboring bacterial cells.

## 1. Introduction

Antimicrobial resistance is regarded as a major threat to human health and has become increasingly severe since the emergence of coronavirus disease 2019 (COVID-19). According to the CDC (Centers for Disease Control and Prevention) report, the number of antibiotic resistant pathogens has increased by at least 15% from 2019–2020 [1]. Among these, carbapenem-resistant *Acinetobacter baumannii* (CRAB) is the most detrimental pathogen because its occurrence rate is the most rapid (35% to over 90%) at hospital onset compared to other pathogens [2]. In addition, hospital-acquired pneumonia and bloodstream infections, the two most prevalent CRAB illnesses, may have a 60% mortality rate [3].

To overcome the problem raised by CRAB, tigecycline (TIG), a last-resort class of tetracycline, has been made commercially available and is used in clinics for the treatment of infections by CRAB [4,5]. Despite its broad clinical use, the continuous use of TIG has accelerated the rate of CRAB acquisition of TIG resistance, rendering human outcomes more hazardous [6,7]. To overcome TIG resistance, the mechanisms and factors involved in TIG resistance should be fully elucidated.

Among the suggested antibiotic resistance mechanisms of AB against ampicillin, carbapenem, polymyxin B (PMB) [8,9,10,11], extracellular vesicles (EVs), and nano-sized (20–400 nm) lipid vesicles carrying almost all biologically functional molecules associated with bacterial communications have been characterized as carriers or decoys for delivering biomolecules that inactivate the functions of antibiotics [12,13,14,15,16,17,18]. Therefore, EV-mediated antibiotic resistance seems to be a common mechanism in AB. However, the involvement of EV in TIG resistance has not been demonstrated. In this study, we aimed to characterize the EV-mediated TIG resistance in AB. Next, we aimed to determine the transferring activity of TIG resistance using TIG-R EVs. Furthermore, we determined the spectrum of EV-mediated TIG resistance in non-AB bacterial cells, and the components of EVs involved in the transfer of TIG-R were characterized. Finally, the efficacy of TIG-R transfer activity between TIG and TIG/TIG-R EV mixtures was compared.

## 2. Materials and Methods

### 2.1. Bacterial Strains, Growth Conditions, and Chemicals

The bacterial strains used in this study are listed in Table 1. Bacterial growth was performed using Luria-Bertani (LB) broth (BD BioSciences, San Jose, CA, USA) with or without 1.5% of Bacto^TM^ Agar (BD BioSciences, San Jose, CA, USA) (named LB-agar) and 37 °C was set as a culture temperature unless indicated otherwise. Tigecycline hydrate and other chemicals used in this study were purchased from Sigma-Aldrich (Sigma-Aldrich, Saint Louis, MO, USA).

### 2.2. MIC Determination

The MIC of tigecycline against bacterial strains were determined using a 96-well plate-based assay, as previously described [19]. One representative from three experiments is shown.

### 2.3. EVs Isolation

EVs from cultures of the ATCC 19606 (TIG-S AB) and TIG-R AB strains were isolated using the column-based ExoBacteria^TM^ OMV Isolation Kit (System Biosciences, Palo Alto, CA, USA), and the final captured EVs on the column were provided with elution buffer in the kit according to the manufacturer’s instructions. EVs were stored at 4 °C before their use.

### 2.4. Characterization of EVs

Both a nanoparticle analyzer (NTA) and transmission electron microscopy (TEM), NanoSight NS300 (Malvern Panalytical, Malvern, UK) and Talos L120C TEM (Thermo Fisher Scientific, Waltham, MA, USA), respectively, were used to characterize the total particle concentration, size distribution, and morphology of EVs without disrupting their structure [20,21]. The protein-based quantification of EVs was performed using Pierce BCA protein assay kit (Thermo Fisher Scientific, Waltham, MA, USA) [22] with a SPECTROStar^®^ Nano (BMG LABTECH GmbH, Ortenberg, Germany). The protein components of EVs were characterized by 12% Mini-PROTEAN® TGX^TM^ Stain-Free gel (Bio-Rad, Hercules, CA, USA) electrophoresis and imaged through the ChemiDoc^TM^ MP Imaging System (Bio-Rad, Hercules, CA, USA) and Image Lab Software (ver.5.2.1, Bio-Rad, Hercules, CA, USA) as described [23]. One representative of the three is shown.

### 2.5. Evaluation of EV-Mediated TIG-R AB Transfer Activity to TIG-S AB and Other Bacteria

To evaluate the transfer activity of TIG-R EVs to AB, bacterial growth (optical density, at 600 nm, OD_600_) of TIG-S AB treated with or without TIG-R EVs (5 μg; BCA quantified) under different concentrations of TIG (0–4 µg·mL^−1^) was monitored every 30 min for 24 h at 37 °C with 500 rpm using a SPECTROStar^®^ Nano (BMG LABTECH GmbH, Ortenberg, Germany). Aliquots (5 μL) from cells grown for 24 h were spotted onto LB-agar plates to determine MIC values for bactericidal activity. The transfer activity of TIG-R EVs from AB to other bacteria was evaluated using a 96-well MIC assay [18]. LB-agar plates or 96-well plates were imaged by ChemiDoc^TM^ MP Imaging System (Bio-Rad, Hercules, CA, USA) and Image Lab Software (ver.5.2.1, Bio-Rad, Hercules, CA, USA). One of representative of the three is shown.

### 2.6. Identification of Components Involved in EV-Mediated TIG Transfer Activity

To identify the role of nucleic acids or proteins in the transfer of EV-mediated TIG-R, TIG-R EVs (5 μg) were treated as previously described [24,25] with minor modifications. Briefly, 2.5 μL of DNase I (1 U∙µL^−1^, Zymo Research, Irvine, CA, USA), *Sau*3AI (4000 U∙mL^−1^, New England Biolabs, Ipswich, MA, USA), or proteinase K (20 mg∙mL^−1^, Thermo Fisher Scientific, Waltham, MA, USA) were treated with TIG-R EVs (5 μg) for 2 h at 37 °C according to the manufacturers’ instruction. The resulting EV fractions were characterized by 1% agarose gel electrophoresis, 12% Mini-PROTEAN® TGX^TM^ Stain-Free gel (Bio-Rad, Hercules, CA, USA) electrophoresis, and imaging analysis. The resulting samples were used to analyze the transference of TIG-R to the TIG-S AB strain.

### 2.7. Evaluation of Recurrence Efficacy of TIG-R by TIG or TIG-R EVs

To determine the efficacy of TIG or TIG-R EVs on recurrent TIG-R, TIG-S cells (ATCC 17978) were initially cultured under different concentrations of TIG. To determine MIC, aliquots from individual samples were spotted on an LB-agar plate, followed by incubation at 37 °C (day 1; passage 0). The next day, the colonies grown at a sub-lethal concentration (0.125 µg·mL^−1^) were further cultured with or without TIG-R EVs (5 µg) under different concentrations of TIG for 16 h in LB broth. This step was performed for 10 days (passage 9) and the recurrence efficacy of TIG or TIG/TIG-R EVs was evaluated. All LB-agar plates were imaged and processed by ChemiDoc^TM^ MP Imaging System (Bio-Rad, Hercules, CA, USA) and Image Lab Software (ver.5.2.1, Bio-Rad, Hercules, CA, USA).

### 2.8. Characterization of EV Proteins

Total EV proteins (10 µg) derived from TIG-S (ATCC 19606) and TIG-R AB were electrophoresed onto 12% Mini-PROTEAN® TGX^TM^ Stain-Free gel (Bio-Rad, Hercules, CA, USA) with Precision Plus Protein^TM^ Standards (Bio-Rad, Hercules, CA, USA). After staining gels with Brilliant Blue R (Sigma-Aldrich, Saint Louis, MO, USA) individual protein bands of new appearances or high intensity in TIG-R EVs compared with those from TIG-S were further subjected to in-gel MALDI-TOF/peptide mass fingerprinting (PMF) analysis using Microflex LRF-20 (Bruker Daltonics, Billerica, MA, USA). Spectra were acquired from 1000 shots per spectrum in the 700–4000 *m/z* range and calibrated by two-point internal calibration using trypsin auto-digestion peaks (*m/z* = 842.5094 and 2211.1040). The peak list was generated using flexAnalysis 3.0 (Bruker Daltonics, Billerica, MA, USA). Finally, the proteins were characterized using the MASCOT search engine (Matrix Science Inc., Boston, MA, USA) and UniProt database ([26]; https://www.uniprot.org; accessed on 13 February 2023).

**Table 1:** Strains used in this study.

Type of Bacteria	Strain ID	Strains	Feature	Reference
Gram- Negative	17978	*Acinetobacter baumannii* (AB)	*Acinetobacter baumannii* (AB) Bouvet and Grimont	ATCC
	19606			
	TIG-R		Laboratory made TIG-R ATCC 19606	[27]
	25922	*Escherichia coli*	Smooth LPS (O6 serotype reference strain)	ATCC
	29906	*Proteus mirabilis*	*Proteus mirabilis* Hauser (Type strain)	ATCC
	14028S	*Salmonella enterica* serovar *typhimurium*	Wild-type *Salmonella enterica* serovar *typhimurium*; a spontaneous mutant resistant to nalidixic acid (NA)	[28]
	16285	*Klebsiella pneumoniae*	*Klebsiella pneumoniae mcr-1* clinical isolates	NCCP
Gram- Positive	25923	*Staphylococcus aureus*	*Staphylococcus aureus* subsp. *aureus* Rosenbach	ATCC

ATCC: American Type Culture Collection (www.atcc.org; accessed on 13 February 2023); NCCP: National Culture Collection for Pathogens (https://nccp.kdca.go.kr/main.do; accessed on 13 February 2023).

### 2.9. Screening of Resistant Antibiotics by TIG-R EVs

Five microliters of nuclease free water (NFW) or TIG-R EVs (5 µg·mL^−1^) were added to 45 μL of Muller Hinton broth (Thermo Fisher Scientific, Waltham, MA, USA) and inoculated with TIG-S (ATCC 19606) AB cells in a Sensititre™ Extended Spectrum Beta-lactamase Plate (Thermo Fisher Scientific, Waltham, MA, USA). The MIC was determined using the micro broth dilution method [19] in accordance with the manufacturer’s instructions.

### 2.10. Statistical Analysis

The biological replicate data are presented as average values with standard deviation. Growth curve data were processed using MARS V4.01 R2 software (BMG Labtech GmbH, Ortenber, Germany) and plotted as average values with standard deviations using SigmaPlot (ver. 12.5) (Systat Software Inc., San Jose, CA, USA).

## 3. Results and Discussion

### 3.1. Physical Characterization of EVs Produced by Tigecycline Resistant A. baumannii

**Figure 1:**
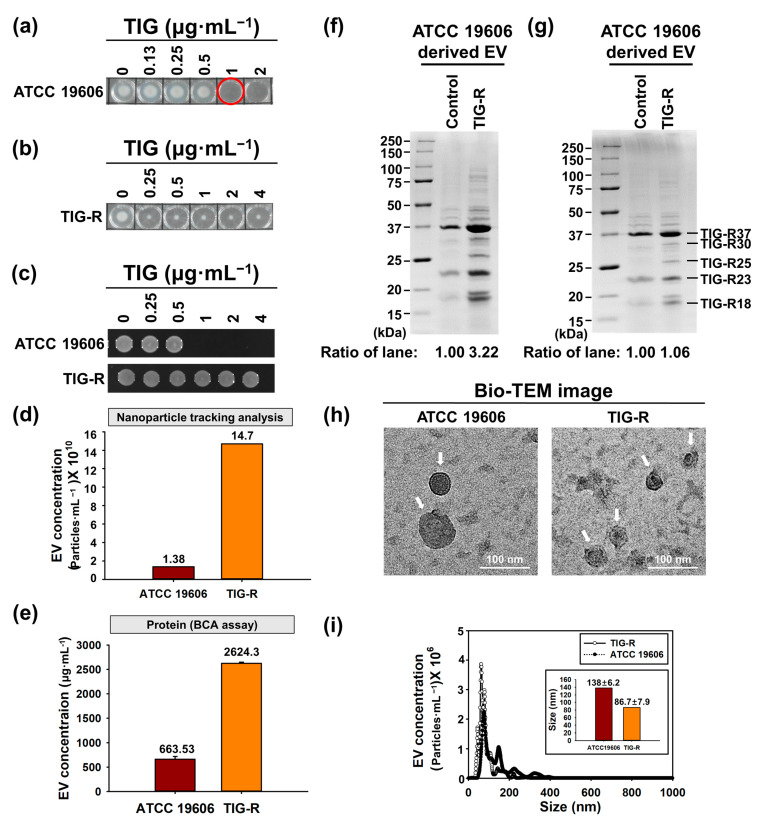
Characterization of strains and EVs produced from ATCC 19606 and TIG-R. (**a**,**b**) MIC determination of TIG-S AB (ATCC 19606) and TIG-R AB strains against TIG (0–4 µg·mL^−1^). Red circle of the plate indicated MIC for TIG. (**c**) Cell viability. Aliquots (5 µL) from (**a**,**b**) were spotted on. EVs were quantified by (**d**) NTA, (**e**) BCA assay (µg·mL^−1^), and protein gel electrophoresis (12% Mini-PROTEAN® TGX^TM^ Stain-Free gel) of EVs with the equivalent amount by (**f**) OD_600_ or (**g**) 10 µg of protein quantified by BCA assay. As a size marker, Precision Plus Protein^TM^ Standards (Bio-Rad, Hercules, CA, USA) was used. The morphology of EVs was analyzed using (**h**) TEM image analysis and the size was determined by (**i**) NTA using the same amount of BCA assay quantified EVs. LB-agar plates and protein bands were imaged by ChemiDoc^TM^ MP Imaging System (Bio-Rad, Hercules, CA, USA) and Image Lab Software (ver.5.2.1, Bio-Rad, Hercules, CA, USA).

EVs from β-lactam antibiotic resistant bacteria, including AB [14], *A. bayli* [29], *Porphyromonas gingivalis* [30], *E. coli* O104:H4 [31], and *S. typhimurium* [32], have been suggested as transfer vehicles of antibiotic resistance to susceptible bacteria via horizontal transfer of the β-lactam resistance gene (*bla_OXA-24_* or *bla_CTX-M-15_*), or via a heterogeneous bacterial community in a non-heritable manner in *E. coli* and methicillin-resistant *S. aureus* (MRSA) [22,33,34]. PMB resistance in *Salmonella* is also induced by EVs from mutant cells, which affect vesiculation [32]. Furthermore, MRSA strains produced a 22.4-fold higher amount of EVs with high defense activity under β-lactam stress [22]. However, the role of EVs in TIG-R AB has not yet been reported. For this purpose, initially, ATCC 19606 and TIG-R AB [27] were selected and their susceptibility status was evaluated by MIC determination, growth curve analysis, and cell viability assays in the presence of TIG as previously described [19]. The latter two methods were chosen to confirm that TIG’s bactericidal activity in MIC is independent of growth rate. Results showed that MIC of TIG against ATCC 19606 and TIG-R AB strains in both methods was 1 and >4 µg·mL^−1^, respectively, for bactericidal activity (Figure 1a–c). Next, EVs from both strains were prepared and their features were characterized using rapid EV quantification methods without disrupting EV structure, which demonstrated that TIG-R AB produced higher numbers of EVs than those from ATCC 19606 (Figure 1d–f), suggesting that TIG-R enhances EV production. Since EVs contain an abundant number of proteins both inside the vesicles and along their membrane as working molecules for the functional activity [35], it is expected that some protein contents either in amount or types from TIG-R EVs will be different. As shown in Figure 1g, five protein bands were identified (named TIG-R18, 23, 25, 30, and 37) as either enriched or solely present in TIG-R EVs. MALDI-TOF/ PMF analysis was conducted to further characterize the identity of the five protein bands in TIG-R EVs, followed by MASCOT and UniProt database analysis to denote the identification and function of the individual proteins shown in Table S1 and Figure S1. The results indicated that the levels of Omp- and porin-associated outer membranes increased in TIG-R EVs. In particular, TIG-R37, denoted as an Omp38 protein (UniProt ID: Q6RYW5), was previously identified as a highly expressed protein in the EVs of AB involved in tetracycline resistance [18], suggesting that the protein induces TIG-R. Next, the size and morphology of EVs from both strains were compared. This revealed that TIG-R EVs were slightly smaller in size with a homogeneous pattern compared with those of ATCC 19606 using TEM (Figure 1h) and NTA analysis (Figure 1i). These results indicated that TIG-R enhanced the production of EVs with smaller sizes and TIG-R-inducing compositions.

### 3.2. TIG-R EV as a Mediator for TIG Resistance

**Figure 2:**
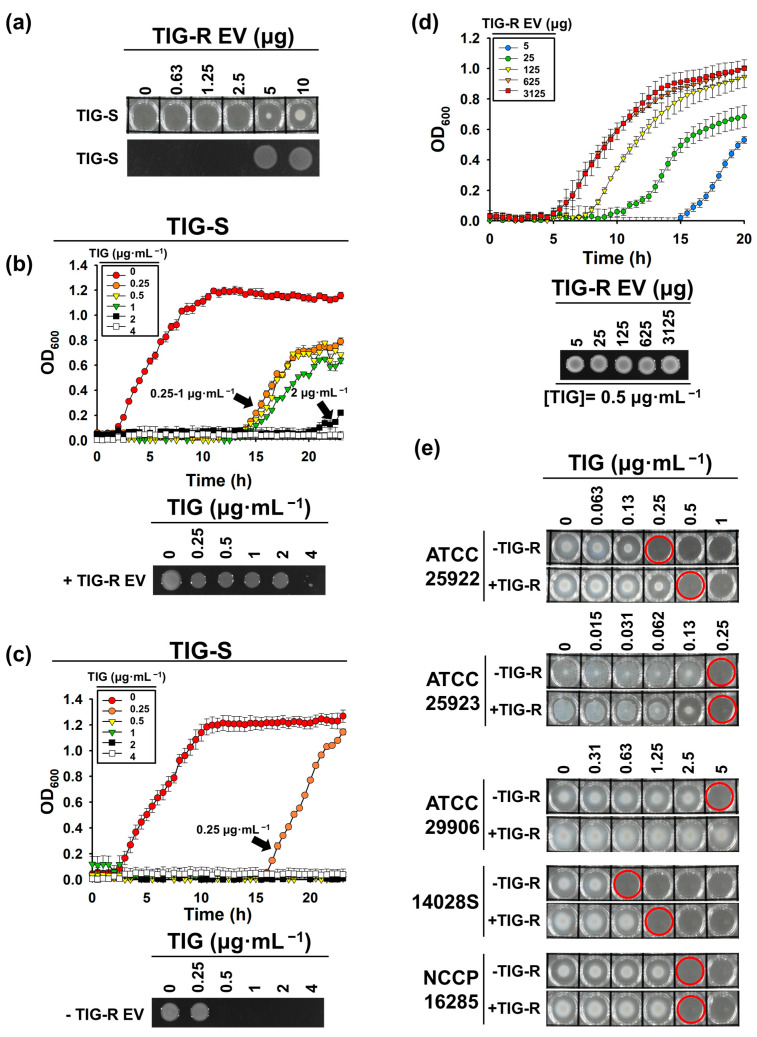
Transfer of TIG-R to AB and other bacterial strains. (**a**) Minimum amount of TIG-R EV inducing TIG-R against TIG-S (ATCC 17978) AB strain. Aliquots (5 µL) treated with TIG-R EVs at indicated concentrations were spotted onto an LB-agar plate containing TIG at 0.5 µg·mL^−1^ and grown for 16 h. Over 5 µg·mL^−1^ of TIG-R EVs, bacterial cells survived on the LB-agar plate. Growth curve analysis of TIG-S AB (ATCC 17978) strain with (**b**, Top) or without (**c**, Top) TIG-R EVs (5 µg) under different concentrations of TIG (0–4 µg·mL^−1^) was performed to evaluate the transfer activity. (**b**,**c**, Bottom) Cell viability. Aliquots (5 µL) of cells from (**b**, Top) and (**c**, Bottom) were spotted onto LB-agar plates and grown for 16 h. LB-agar plates were imaged by ChemiDoc^TM^ MP Imaging System (Bio-Rad, Hercules, CA, USA) and Image Lab Software (ver.5.2.1, Bio-Rad, Hercules, CA, USA). Error bars in growth curves indicate statistical significance at *p* < 0.05 (n = 3). (**d**) Dose-dependency of TIG resistance. Growth curve of TIG-S AB strain was conducted under TIG at 0.5 µg·mL^−1^ with increasing amounts of TIG-R EVs. Cells were grown at 37 °C for 24 h with 500 rpm in 96-well microplates and growth was monitored by measuring the OD_600_ using a SPECTROStar*^®^* Nano (BMG LABTECH GmbH, Ortenberg, Germany, n = 3). (**e**) Transfer spectrum of TIG-R EVs. TIG MICs of *E. coli* (ATCC 25922), *K. pneumoniae* (NCCP 16285), *P. mirabilis* (ATCC 29906), *S. aureus* (ATCC 25923), and *S. typhimurium* (14028S) against TIG (0–5 µg·mL^−1^) without or with TIG-R EVs of 5 µg was determined. One of the representative data from n = 3 was shown. The 96-well plates were imaged with a digital camera (Samsung NX200, Suwon, Korea). Red circles indicate MICs for individual samples.

To determine whether TIG-R EV is a vehicle for the transfer of TIG-R, TIG-R EVs (0–5 μg, BCA quantified) were incubated with ATCC 17978 (TIG-S AB) cells for 24 h under the presence of TIG at 0.5 µg·mL^−1^. The data revealed that the minimum amount of TIG-R EV expressing TIG-R was 5 μg less than that in our method (Figure 2a). Growth curve and cell viability analyses were performed to characterize the role of TIG-R EVs in the development of TIG-R. We observed that TIG-S AB strain treated with or without TIG-R EVs exhibited the MIC of TIG at 4 or 0.5 µg·mL^−1^, respectively (Figure 2b,c). Next, dose-dependency of EV-mediated TIG-R transfer was assessed against the TIG-S AB strain using growth curve determination under 5–3125 μg of TIG-R EVs. Results revealed that the more the EVs were applied, the less the bacterial growth retardation was after 6 h between 5 and 3125 μg EVs, as measured by turbidity (OD _600_) (Figure 2d). This suggests that EV can transfer TIG-R via two modes of action: direct recognition and transfer of resistance. Moreover, the action seems to be a group behavior, in which individual EVs can help each other to transfer resistance to susceptible bacteria for a rapid increase in resistant populations. In a previous report, EVs from β-lactam resistant bacteria could transfer their resistance to other bacterial species [22,34]. Therefore, the spectrum of TIG-R EV-mediated resistance was assessed against different gram-negative and positive strains in the presence of TIG-R EV (5 μg) derived from TIG-R AB strain (Figure 2e). Results showed that the MIC of *E. coli*, *P. mirabilis*, and *S. typhimurium* exhibited a 2-fold increase in resistance compared with non-TIG-R EV-treated cells. However, the TIG-R EVs did not affect the susceptibility of *K. pneumonia* and *S. aureus* to TIG. Therefore, TIG-R AB-derived EV can transfer TIG resistance to neighboring gram-negative bacteria including *E. coli*, *P. mirabilis*, and *S. typhimurium* strains. However, the exact mechanisms underlying this selectivity remain unclear.

### 3.3. Proteins Are Major Determinants for EV-Mediated TIG-R Transfer

**Figure 3:**
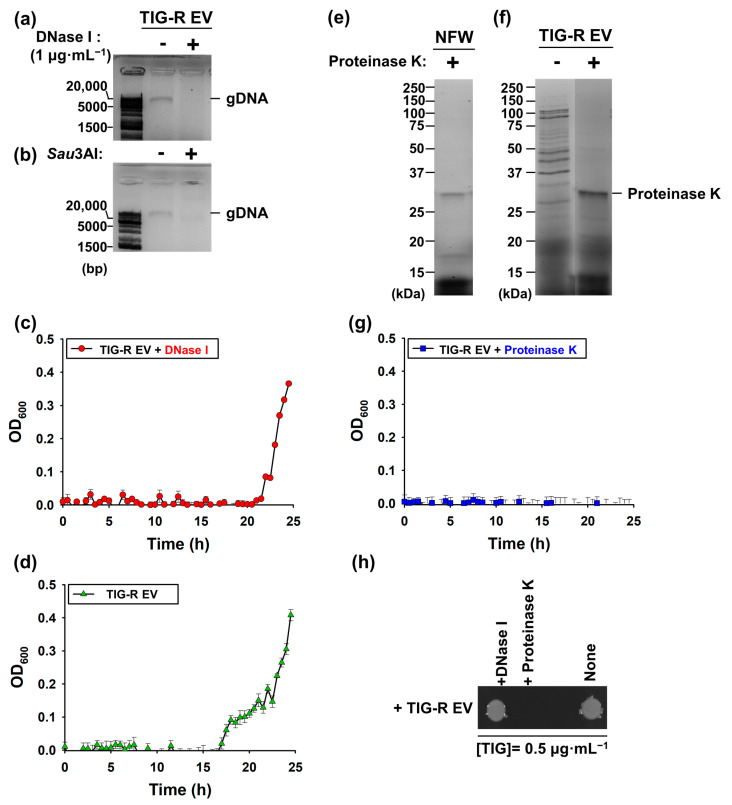
Identification of determinants for the TIG transfer activity. Degradation of DNA components by DNase or restriction enzyme. TIG-R EV components. TIG-R EVs were treated with (**a**) DNase I and (**b**) *Sau*3AI. TIG-R EVs added with DNase I, *Sau*3AI were observed by 1% agarose gel electrophoresis with 1 kb Plus DNA Ladder (Thermo Fisher Scientific, Waltham, MA, USA). Growth curve analysis of TIG-S AB (ATCC 17978) strain with TIG-R EVs (5 µg) pre-treated with (**c**) DNase I (Red, circle), (**d**) NFW (Green, up-triangle), and (**g**) proteinase K (Blue, square) under TIG (0.5 µg·mL^−1^). Cell growth (OD_600_) was monitored by SPECTROStar*^®^* Nano (BMG LABTECH GmbH, Ortenberg, Germany, n = 3). (**e**,**f**) Characterization of proteins from TIG-R EVs after proteinase K treatment. EV proteins and proteinase K were identified using 12% Mini-PROTEAN® TGX^TM^ Stain-Free gels (Bio-Rad, Hercules, CA, USA) with Precision Plus Protein^TM^ Standards (Bio-Rad, Hercules, CA, USA). Images (**a**,**b**,**e**,**f**) were obtained by ChemiDoc^TM^ MP Imaging System (Bio-Rad, Hercules, CA, USA) and Image Lab Software (ver.5.2.1, Bio-Rad, Hercules, CA, USA). (**h**) Cell viability. Aliquots of cells from (**c**,**d**,**g**) were spotted after growth for 16 h. All images (**a**,**b**,**e**,**f**,**h**) were obtained by ChemiDoc^TM^ MP Imaging System (Bio-Rad, Hercules, CA, USA) and Image Lab Software (ver.5.2.1, Bio-Rad, Hercules, CA, USA).

EVs contain bioactive molecules [36,37]. Horizontal transfer of DNA components is a known mechanism of AB resistance [38,39]. To test whether the DNA components in TIG-R EVs are major factors for TIG-R, the EVs were initially treated with DNase I, a non-specific DNase to eliminate DNA components in EVs, and it was found that DNA ~2 kb in size was completely degraded (Figure 3a). This DNA band was also eliminated using the additional restriction enzyme *Sau*3AI (Figure 3b). Next, the transfer activity of TIG-EV (5 μg) treated with DNase I was applied to TIG-S cells and the effect was analyzed by both growth curve determination and cell viability under 0.5 µg·mL^−1^ of TIG. The results showed that DNase I-treated TIG-R EVs retained the transfer activity of TIG as non-treated TIG-R EVs (Figure 3c,d). These results indicated that horizontal transfer of DNA was not the major mechanism for transferring TIG resistance to TIG-R EVs. As proteins are another major component of EVs and can cause antibiotic resistance [15], the effect of protein-deficient TIG-R EVs on transfer activity was analyzed. To this end, TIG-R EVs were treated with proteinase K, a serine protease (28.9 kDa in size) that cleaves wide range of protein substrates with high efficiency, which was located in size markers between 25 and 37 kDa on a protein gel (Figure 3e), and it was found that major protein components were eliminated in EV (Figure 3f). Using proteinase K-treated TIG-R EVs, the transfer activity of TIG was assessed under the same conditions as the DNase-treated EV. As shown in Figure 3g, the transfer activity of the EV completely disappeared compared to that of non-proteinase K-treated TIG-R EVs. Additionally, neither proteinase K nor DNase I alone affected bacterial survival rate. Overall, it was confirmed that the protein components of TIG-R EVs were the major factors involved in EV-mediated TIG-R transfer.

### 3.4. Comparison of EV-Mediated Transfer of Resistance to Antibiotics

**Figure 4:**
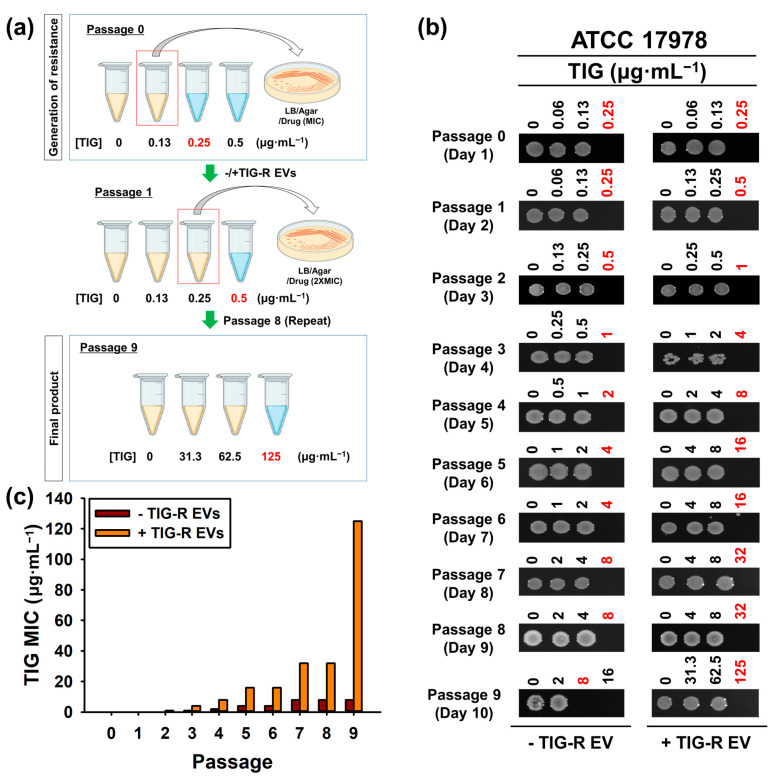
Evaluation of recurrence by TIG and TIG-R EVs. (**a**) Schematic representation of resistance generation method. (**b**) Determination of MIC values. Aliquots (5 µL) from cells at different concentrations of TIG from each passage were spotted onto LB-agar plates and were grown for 16 h. LB-agar plates were imaged and further processed by ChemiDoc^TM^ MP Imaging System (Bio-Rad, Hercules, CA, USA) and Image Lab Software (ver.5.2.1, Bio-Rad, Hercules, CA, USA). One representative from n = 3 was shown. Red indicates MIC values for individual passage cells. (**c**) Comparison of TIG MIC between cells from (**b**).

In this study, we showed that EVs mediate TIG-R expression in a dose-dependent manner (Figure 2). However, the role of TIG-R EVs in the evolution of TIG has not yet been investigated. To this end, TIG-S AB (ATCC 17978) cells were consecutively treated with TIG-R EVs (5 μg) or TIG under sub-lethal concentrations of TIG for 10 days and the MIC of TIG from the samples was determined (Figure 4a). The results demonstrated that the MIC of TIG with the addition of original TIG-R EVs after 10 days (passage 9) showed an increase in TIG resistance to 512-fold, when it compared with original TIG-S AB strain (Day 1; passage 0), whereas the consecutive exposure of TIG itself to TIG-S AB strain acquired TIG resistance to a 32-fold maximum (Figure 4b,c). These results indicate that TIG-R EVs can act as biomaterials that strongly induce TIG-R compared with the antibiotic itself.

### 3.5. Role of TIG-R EVs on Current Antibiotics

Multidrug resistance (MDR) could be induced by a single antibiotic if the drugs share the same mechanism [40]. In addition, the TIG-R EV contained a resistance protein associated with carbapenems and tigecycline (Table S1). These results indicate that TIG-R EVs can induce MDR in AB. To identify the role of TIG-R EVs as specific materials that induce antibiotic-associated MDR, profiling of antibiotic spectrums under TIG-R EVs (5 µg·mL^−1^) against AB was determined using a pre-made Sensititre™ Extended Spectrum Beta-lactamase MIC Plate (Figure S2a). The results showed that TIG-R EVs induced resistance to cefpodoxime, cefotaxime, ampicillin, piperacillin/tazobactam constant 4, cefepime, and cefoxitin, compared with the control set (Figure S2b,c). Among them, cefpodoxime and piperacillin/tazobactam constant 4 expressed the resistance to TIG-S more than 8-fold. Notably, most of selected antibiotics, except piperacillin/tazobactam constant 4, which is a β-lactamase inhibitor broad-spectrum antibiotic, are known to work as inhibitors of bacterial cell wall synthesis. This might be affected by changing the level of Omp, an outer membrane porin protein (Table S1; [41]), and carbapenem-resistant protein (Table S1 and Figure S1), suggesting that TIG-R EVs are key enhancers of CRAB populations.

## 4. Conclusions

In this study, we report a new and uncharacterized mechanism for TIG-R AB or CRAB, wherein TIG-R resistance proteins are transferred via TIG-R AB-derived EVs with a high recurrence rate compared with TIG itself. Moreover, TIG-R EVs from AB can selectively transfer their resistance to other gram-negative bacteria, except *K. pneumoniae*. Our results provide future perspectives for overcoming the rapid increase in MDR in gram-negative bacterial populations. These include: (1) how broadly this mechanism can be applied to clinical isolates and other antibiotic-resistant bacteria, (2) how to eliminate EV production, and (3) which specific factors should be eliminated. As TIG is a widely used antibiotic against gram-negative bacterial infections, research can provide concrete evidence that eliminating EVs from TIG-R significantly decreases MDR gram-negative bacterial populations, including CRAB. However, our work has several limitations that must be addressed in the future. Firstly, we did not identify any specific proteins involved in TIG-R transfer, though we did suggest some potential TIG-R proteins. This is possible through EV proteomics and phenotypic characterization of individual EV-enriched proteins via ectopic protein expression and MIC determination. Secondly, there is no clear mechanism for how EV transferred TIG-R to susceptible bacteria. TIG-R EVs and gene knockout libraries can be used to identify universal genes involved in EV transfer activity against *A. baumannii* and other gram-negative bacteria, apart from *K. pneumoniae*. We may also be able to explain why TIG-R EVs from *A. baumannii* are unable to transfer resistance to gram-positive bacteria with this information. Thirdly, because our study used *A. baumannii* with only TIG-R, it is unclear whether TIG-R EVs can be selectively transferred to susceptible bacteria when TIG-R EVs are derived from clinical MDR gram-negative pathogens with multiple types of antibiotic resistance. This question could be answered by examining the transfer efficiency of individual antibiotic resistance, as described in this study.

## Data Availability

The data presented in this study are available from the corresponding author upon reasonable request.

## Supplementary Materials

The following supporting information can be downloaded at: https://www.mdpi.com/article/10.3390/pharmaceutics15041251/s1, Table S1: MALDI-TOF/MS analysis of TIG-R EVs fraction.; Figure S1: Characterization of TIG-R derived EVs; Figure S2: Confirmation of TIG-R EV-mediated resistance to other antibiotics.
