# Supplementary material for: Characterization of Increased Extracellular Vesicle-Mediated Tigecycline Resistance in Acinetobacter baumannii
Source: Pharmaceutics. 2023 Apr 15;15(4):1251. doi: 10.3390/pharmaceutics15041251 (PMC10146562; doi:10.3390/pharmaceutics15041251)
Supplement: Supplementary file 1 [file pharmaceutics-15-01251-s001.zip › pharmaceutics-2250637-supplementary.pdf]

Supplementary Materials: Characterization of increased extracellular vesicle-mediated tigecycline resistance in *Acinetobacter baumannii*

Hyejin Cho, Tesalonika Sondak, and Kwang-sun Kim*

Department of Chemistry and Chemistry Institute of Functional Materials, Pusan National University, Busan, 46241, South Korea

*Correspondence: kwangsun.kim@pusan.ac.kr; Tel.: +82-51-510-2241

(a) TIG-R18

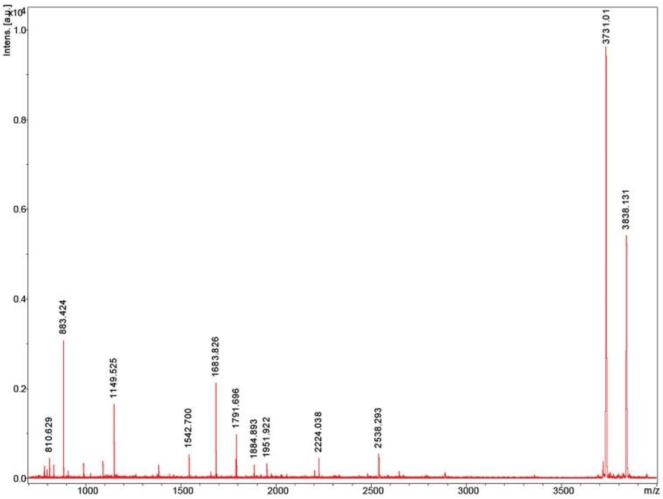

1 MKNIQKSLLA ALIVAGYAVN TQAAVTGQVD VKLNISTGCT VGGSQTEGNM 50

51 NK**FGTLNFGK** TSGTWNNVLT AEVASAATGG NISVTCDGTD PVDFTVAIDG 100

101 GERTDR**TLKN** **TASADVVAYN** **VYR**DAAR**TNL** **YVVNQPQQFT** **TVSGQATAVP** 150

151 **IFGAIAPNTG** **TPKAQGDYKD** **TLLVTVNF** 178

(b) TIG-R23

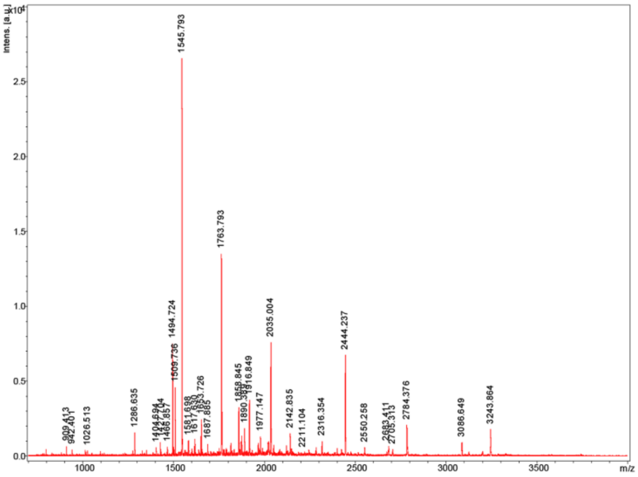

1 MKKLAIASAL LSALAVSGAA NAYQAEVGGS YNYLDPDNGS SVSK**FGVDGT** 50

51 **YYFNPVQTRN** **APLAEAAFLN** **R**ASNVNAHVN YGDNSGTK**DT** **QYGVGVEYFV** 100

101 **PNSDFYLSGD** **VGR**NEREIDN TNIDSK**VTTY** **AAEVGYLPAP** **GLLLALGVK**G 150

151 YDEKDGKDGA DPTVRAK**YVT** **QVGQHDVNLE** **AYGAFGDLDE** **YKVR**GDYYID 200

201 K**TLSLGVDYY** **NNDLTDKDEF** **GINAKKFLNQ** **QVSVEGR**VGF GDNDNTYGVR 250

251 AAYRF 255

(c) TIG-R25

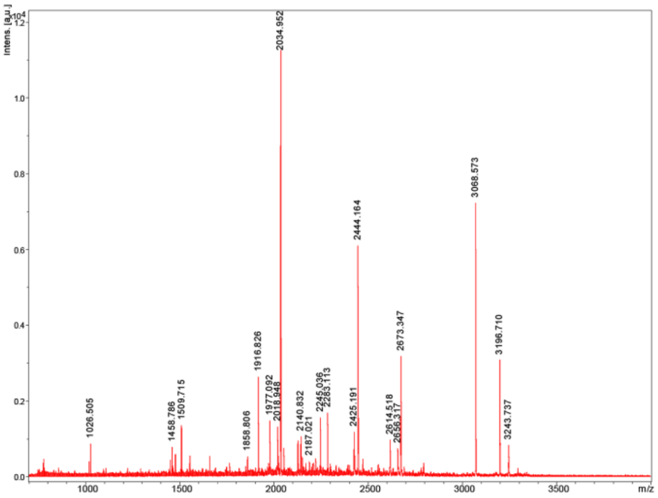

1 MKVLRVLVTT TALLAAGAAM ADEAVVHDSY AFDKNQLIPV GARAEVGTTG 50

51 YGGALLWQAN PYVGLALGYN GGDISWTDDV SVNGTKYDLD MDNNNVYLNA 100

101 EIRPWGASTN PWAQGLYIAA GAAYLDNDYD LAKR**IGNGDT** **LSIDGKNYQQ** 150

151 **AVPGQEGGVR** **GKMSYKNDIA** **PYLGFGFAPK** **ISKNWGVFGE** **VGAYYTGNPK** 200

201 **VELTQYNLAP** **VTGNPTSAQD** **AVDKEANEIR** NDNKYEWMPV GKVGVNFYW 249

(d) TIG-R30

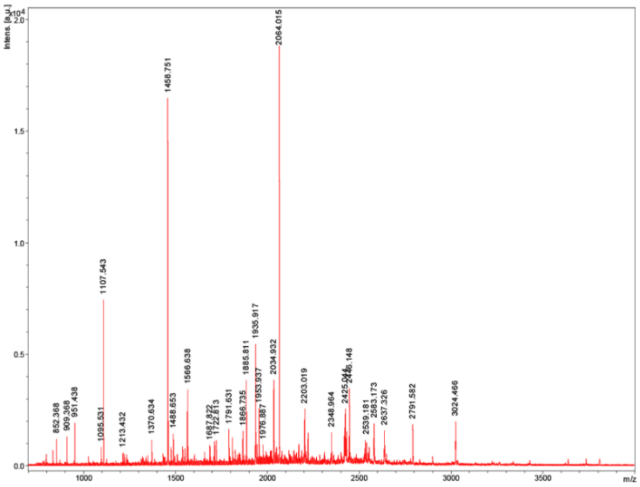

1 **MRFSQLK**VGV IAALLSVSSF AAQEFLNVSY DPTRELYTDF NK**QFGTYWKQ** 50

51 RTGQDIEFKQ SHGGSGKQAR **AVIDGLNADV** **VTLALAADID** **EIAEK**AKLLP 100

101 TDWQKK**LPQN** **STPYTSTIVF** **LVRK**GNPKQI KDWGDLIKPG VEIITPNPKT 150

151 SGGARWNYLA AWAWAKHQAG GNDAKAQEFV RQIYKHTKVL DSGARGATTT 200

201 FAER**GIGDVL** **LAWENEAHLA** **IREQPGKFEI** **VTPSLSILAE** **PPVAIVEK**NA 250

251 AKK**GNLTIAK** **GYLNYLYSPA** **GQEIAARNFY** **RPR**NAAVLK**K** **YSNVFKPLK**L 300

301 VTIDKEFGGW TKVQKQHFDN GGVFDQIVKI NSAEK 335

(e) TIG-R37

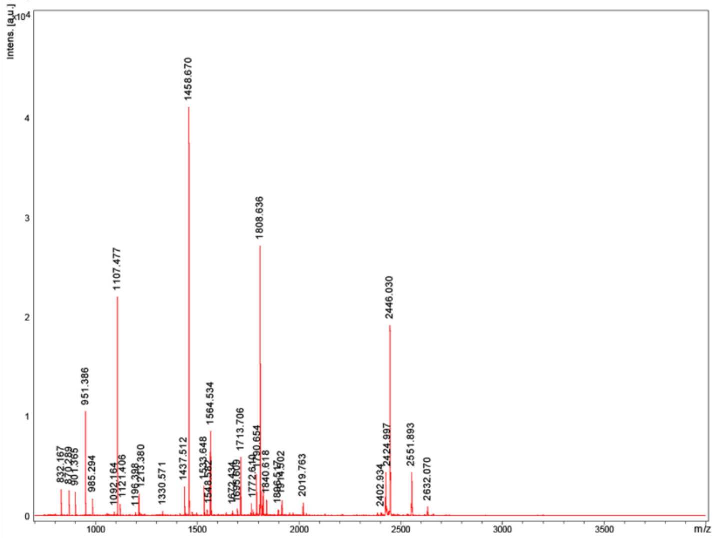

1 MKLSRIALAT MLVAAPLAAA NAGVTVTPLL LGYTFQDSQH NNGGKDGNLT 50

51 NGPELQDDLF VGAALGIELT PWLGFEAEYN QVKGDVDGAS AGAEYKQKQI 100

101 NGNFYVTSDL ITKNYDSK**IK** **PYVLLGAGHY** **KYDFDGVNR**G TRGTSEEGTL 150

151 GNAGVGAFWR **LNDALSLR**TE ARATYNADEE FWNYTALAGL NVVLGGHLKP 200

201 AAPVVEVAPV EPTPVAPQPQ ELTEDLNMEL R**VFFDTNKSN** **IKDQYKPEIA** 250

251 **K**VAEK**LSEYP** **NATARIEGHT** **DNTGPR**KLNE RLSLARANSV K**SALVNEYNV** 300

301 **DASR**LSTQGF AWDQPIADNK TKEGRAMNR**R** **VFATITGSR**T VVVQPGQEAA 350

351 APAAAQ 356

Figure S1. MALDI-TOF/MS spectrum and amino acid sequences of protein bands from TIG-R derived EVs. Mass spectral data (top) and full amino acids to matched proteins (bottom) of (**a**) TIG-R 18, (**b**) TIG-R23, (**c**) TIG-R25, (**d**) TIG-R30, and (**e**) TIG-R37 are shown. Band IDs are adapted from **Figure 1**. All analysis of peptide fragments were as described in *Materials and Methods*. Bold underlined sequences below the mass spectrum indicate matched amino acids to the proteins adapted from UniProt database ([26]; www.uniprot.org; accessed on February 2023).

(a)

(µg·mL⁻¹)	1	2	3	4	5	6	7	8	9	10	11	12
A	AXO								MERO			
	1	2	4	8	16	32	64	128	1	2	4	8
B	CEP		POD								CIP	
	8	16	0.25	0.5	1	2	4	8	16	32	1	2
C	FOT									GEN		
	0.25	0.5	1	2	4	8	16	32	64	4	8	16
D	F/C										AMP	
	0.12/4	0.25/4	0.5/4	1/4	2/4	4/4	8/4	16/4	32/4	64/4	8	16
E	TAZ										FAZ	
	0.25	0.5	1	2	4	8	16	32	64	128	8	16
F	T/C											POS
	0.12/4	0.25/4	0.5/4	1/4	2/4	4/4	8/4	16/4	32/4	64/4	128/4	
G	IMI						P/T4					
	0.5	1	2	4	8	16	4/4	8/4	16/4	32/4	64/4	
H	FEP					FOX					NEG	
	1	2	4	8	16	4	8	16	32	64		

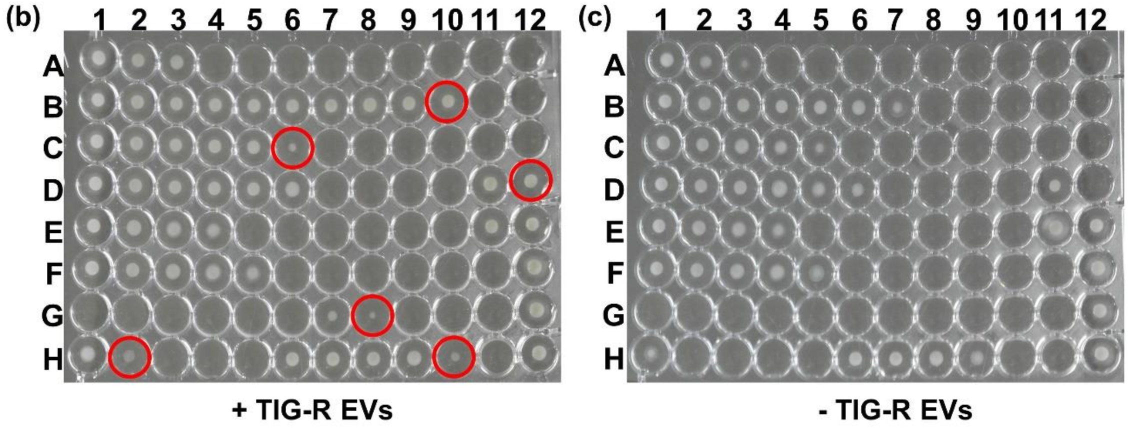

Figure S2. Confirmation of TIG-R EV-mediated resistance to other antibiotics. (**a**) Information on antibiotics and the working concentration. Sensititre™ Extended Spectrum Beta-lactamase Plate (Cat. No. ESB1F, Thermo Fisher Scientific, Waltham, MA, USA) with final concentration of individual antibiotics shown. TAZ, Ceftazidime; FAZ, Cefazolin; FEP, Cefepime; FOX, Cefoxitin; CEP, Cephalothin; POD, Cefpodoxime; FOT, Cefotaxime; AXO, Ceftriaxone; IMI, Imipenem; MERO, Meropenem; GEN, Gentamicin; AMP, Ampicillin; CIP, Ciprofloxacin; P/T4, Piperacillin/tazobactam constant 4; T/C, Ceftazidime/clavulanic acid; F/C, Cefotaxime/clavulanic acid; NEG and POS indicate the negative and positive control, respectively. (**b**, **c**) Effect of TIG-R EVs on the resistance to antibiotics. TIG-S cells were cultured in a Sensititre™ Extended Spectrum Beta-lactamase Plate with (**b**) or without (**c**) TIG-R EVs (5 µg·mL⁻¹). Red circle of the plate indicated resistance for each antibiotic. One representative from n = 3 was shown. The 96-well plates were imaged with digital camera (Samsung NX200, Suwon, Korea).

Table S1. Identification of proteins enriched or solely presented in TIG-R EVs

Band ID	Accession No.	Protein size (amino acids)	Coverage to proteins (%)	Function
TIG-R37	Q6RYW5	356	26	Outer membrane protein
TIG-R30	D0CBP2	335	42	Sulfate ABC transporter periplasmic substrate-binding protein
TIG-R25	D0CBN6	249	37	Carbapenem-associated resistance protein
TIG-R23	D0CF50	255	54	General bacterial porin
TIG-R18	A0A6F8TDQ5	178	42	Spore Coat Protein U domain protein

Band ID indicates the protein bands shown in **Figure 1**. Based on the Mass spectral data from **Figure S1** and searches of MASCOT database and UniProt database ([26]; www.uniprot.com; accessed on Fe bruary 13, 2023), proteins were identified as encoded from *Acinetobacter baumannii*. Coverage (%) was determined as the percentile of peptides overlapping to full-size proteins.

Reference

26. The UniProt Consortium. UniProt: The Universal Protein Knowledgebase in 2023. *Nucleic Acids Res.* **2023**, *51*, D523–D531. https://doi.org/10.1093/nar/gkac1052.
